# Active Packaging for the Extended Shelf-Life of Meat: Perspectives from Consumption Habits, Market Requirements and Packaging Practices in China and New Zealand

**DOI:** 10.3390/foods11182903

**Published:** 2022-09-19

**Authors:** Xin Li, Renyu Zhang, Mohammad Mahbubul Hassan, Zhe Cheng, John Mills, Chengli Hou, Carolina E. Realini, Li Chen, Li Day, Xiaochun Zheng, Dequan Zhang, Talia M. Hicks

**Affiliations:** 1 Institute of Food Science and Technology, Chinese Academy of Agricultural Sciences/Key Laboratory of Agro-Products Quality & Safety in Harvest, Storage, Transportation, Management and Control, Ministry of Agriculture and Rural Affairs, Beijing 100193, China; 2 Food Technology & Processing Team, AgResearch Ltd., Te Ohu Rangahau Kai, Palmerston North 4474, New Zealand; 3 Fashion, Textiles and Technology Institute, University of the Arts London, London EC2A 3AA, UK; 4 Food System Integrity Team, AgResearch Ltd., Hopkirk Research Institute, Palmerston North 4442, New Zealand; 5 Food & Fibre Off-Farm Sector, AgResearch Ltd., Te Ohu Rangahau Kai, Palmerston North 4422, New Zealand

**Keywords:** active packaging, meat, sustainable strategies, processing optimization, packaging manufacture, legislation

## Abstract

Active packaging (AP) has been developed to improve the safety, quality and integrity of food, and minimise food waste, while its application in meat is scarce. This review aims to describe meat production and consumption culture in China and New Zealand to provide the context for packaging innovation requirements, focusing on the emerging opportunities for AP to be used for the improvement of the shelf-life of pre-rigor, aged, and frozen-thawed meat products. Sustainable polymers utilised in the manufacturing of AP, manufacturing techniques, the release mechanisms of actives, and legal and regulatory constraints are also discussed. Diverse market compositions and consumption cultures in China and New Zealand require different packaging solutions to extend the shelf-life of meat. AP containing antimicrobials, moisture regulating agents, and antioxidants may be used for pre-rigor, dry- and wet-aged products and in improving the quality and shelf-life of frozen-thawed meat. Further innovations using sustainably produced polymers for AP, along with incorporating active compounds of multiple functions for effectively improving meat quality and shelf-life are necessary. Challenges remain to resolve issues with scaling the technology to commercially relevant volumes as well as complying with the rigorous legal and regulatory constraints in various countries.

## 1. Introduction

Meat is a valuable food product, rich in essential proteins, lipids and micronutrients in forms that are readily digestible which often provide nutritional adequacy for a wide range of people. However, the nutritional composition and high-moisture content of meat also make it susceptible to deterioration in quality, arising from microbial spoilage and other enzymatic and oxidative deterioration [1,2]. Losses and waste along the meat supply chain represent between 21–30% of the total global production volume, with around half occurring during the distribution, retail and consumer use [3]. Given the environmental cost of rearing livestock for producing meat and the benefits yielded by its consumption, it is vital that meat products are properly packaged to improve their shelf-life during distribution, retail, and consumption.

The primary purpose of meat packaging is to provide a physical barrier that will protect the meat product from its surrounding environment, while also limiting microbial growth and oxidation, thereby extending the product’s shelf-life [4]. However, most contemporary packaging materials used in the industry are challenging to recycle through mechanical or chemical processes and can often accumulate in the environment. Hence researchers and responsible industries have begun to consider sustainable strategies through packaging innovations and product stewardship to reduce the overall environmental impact of meat packaging, while still supplying consumers with meat products of consistent and sustained quality. In the last few decades, scientific research has been focused on developing more eco-friendly and sustainable packaging materials from renewable resources that are compostable/fully biodegradable, or even edible [2]; much of which has been focussed on the application of these novel packaging materials through a variety of packaging formats in maintaining or improving the quality and extending the shelf-life of meat products [5,6,7].

Active packaging (AP) is a relatively new type of packaging which contains various active compounds, such as antioxidants, antimicrobials, moisture and various gas absorbers, and ultraviolet radiation absorbers, that interact with the packaged food or the surrounding environment to extend shelf-life by maintaining the quality, safety, and integrity of food. The active compounds from the package further increase the shelf-life of food products over and above conventional packaging by limiting the deleterious effects caused by oxidation, microbial growth, and moisture loss during storage [8].

The application of AP in the food industry has been suggested as a strategy with the potential to contribute holistically to mitigate global warming, reduce fossil-energy demand and decrease acidification and eutrophication potential [9]. However, whether AP can be tailored as a sustainable approach to improve the efficiency during the processing of value-added meat products remains unexplored. Further, the distinct meat production systems and market composition in different countries could also play a key role in defining the needs for packaging solutions. For instance, China is one of the major meat producers and consumers, representing a unique food culture in the eastern world where packaging solutions would focus on the requirements from the domestic market, whereas New Zealand (NZ) exports most of its fresh and frozen beef and lamb, with packaging requirements being focused on preserving quality for its export markets, including China.

Recently, there have been a number of reviews published that comprehensively summarise the types of AP and their applications for preserving or even enhancing the quality and safety of food products during storage [2,10,11,12,13,14]. However, little has been reviewed for meat products, specifically on AP requirements based on meat consumption cultures and market requirements. The current review is focused on the Chinese and NZ markets as case studies for understanding the role of food culture and market requirements on the development of meat packaging solutions. Further, it explores how AP may apply in the optimisation of current processing regimes for dry- and wet-aged meat and improving the quality of pre-rigor meat, frozen and thawed products. This article also provides an overview of the manufacturing techniques and legislations for AP materials. Finally, some concerns and pitfalls on the application of AP and future areas of innovation are discussed.

## 2. Production and Market Composition of Meat in China and NZ

Meat is produced and marketed in a variety of formats and generally categorised as unprocessed or processed meat. Depending on the market, it is possible for a consumer to purchase meat pre-rigor (hot meat), post-rigor (aged meat) and after further processing (curing, drying, marinating, smoking, and cooking). As such, packaging requirements differ depending on the type of meat products, route to market and target consumers, while maintaining the safety and quality of the product and minimising packaging costs.

### 2.1. Meat Market Needs in China and NZ

#### 2.1.1. Meat Production and Market Composition

China is one of the most rapidly developing countries, with increasing demands for meat and meat products. As a result, China has become one of the largest countries in meat production and consumption worldwide. In 2021, China’s annual output of meat was 89.9 million tonnes (MT), of which pork was 53.0 MT, beef and veal was 7.0 MT, lamb and mutton was 5.1 MT and poultry was 23.8 MT [15]. Most of the meat produced in China is consumed domestically, however, China also imports a significant amount of meat to satisfy the rising demands for meat and meat products. As of 2021, China imported 2.3 MT of beef, 410.6 thousand tonnes (TT) of lamb and mutton. AP could be an effective packaging strategy for maintaining the quality of meat that produced domestically or imported.

New Zealand is one of the largest global producers and exporters of lamb and venison, and a minor global producer of beef and pork. In 2021, NZ produced 454 TT of lamb and mutton, 759 TT of beef and veal, 44.9 TT of pork and 10.7 TT of venison [16]. The domestic red meat market (mainly beef and lamb) in NZ is small, therefore most of the production is exported, except for pork. The export of NZ meat is dominated by beef (48%) and lamb (44%) followed by other meat products (8%). As of 2022, about 96% of lamb, 86% of mutton, and 87% of beef and veal meat will be exported from NZ to more than 120 countries [17]. The majority of exports were in sub-primal form rather than carcasses, with over 98% of lamb, 35% of mutton and 99% of beef exported as sub-primal cuts. In addition, about 27% of NZ lamb is exported as a high value chilled product and the rest as a traditional frozen product. Given the fact that most meat products produced in NZ are exported, the application of AP to maintain the quality and integrity of chilled meats would be aimed for export markets.

#### 2.1.2. Consumption Culture

For thousands of years, the unique dietary habits of Chinese people have gradually coalesced to become an important part of traditional Chinese culture. Chinese foods are artistic and versatile, which means that the same food can be prepared differently depending on the region, season, and consumer, leading to variations in tastes. Traditionally, fresh meat is mostly prepared for stir-frying, stewing and braising. With the development of the economy in China over the last two decades, food consumption is not only for satiety, but also for sensorial and psychological satisfaction [18]. The daily meal structure is changing toward a high protein content, derived more from animal origin rather than those of traditional plant and grain-based diets. It is reported that the meat consumption in China accounts for a quarter of the total global meat consumption and consumers are more inclined to purchase fresh meat for domestic processing. The emphasis and desire for ‘freshness’ have driven the prominence of meat that comes from animals that are slaughtered earlier in the day [19]. This type of meat is known as ‘hot meat’ (i.e., pre-rigor meat). Therefore, daily shopping for fresh meat and other food ingredients is a common practice in Chinese households.

The globalisation of markets and the introduction of western and other Asian foods and cuisine in China are altering consumer eating behaviours of meat and meat products [19]. Consumers nowadays have become more accepting of steakhouse-type restaurants. Fresh meat products imported from countries like NZ, Australia, Spain, and the US are available for purchase from online stores and local supermarkets. Economic development in China has been accompanied by an increase in expendable income, and has resulted in consumers becoming more health-conscious and discerning of higher quality meat products, while also becoming more concerned about product safety and authenticity [19]. There is a growing demand for chilled compared to frozen meat in China due to the association of chilled meat being fresher and of higher quality than frozen meat.

NZ is a multicultural country with immigrants originating from all around the world. Food culture in NZ is represented by western food which considers food as the origin of nutrition and energy, therefore animal-derived proteins such as those from meat and dairy products, are the focal point of the meal and vegetables are ancillary. Consumption of poultry, beef, and pork are the most popular, accounting for 96% of meat consumption in NZ. Fresh meat, which has been chilled and/or aged, is widely available in butcher stores and supermarkets and preferred by consumers over frozen meat; with grilling/braising, frying, roasting and stewing being the most common cooking methods. In addition, processed meat products, such as sausages, salami, ham, bacon and smoked meat are also high in demand, with 41.8% of New Zealanders eating processed meat one to two times per week, and 28.9% eating processed meat three or more times per week [20].

### 2.2. Current Packaging Formats Used in Retail and Export Markets

The packaging requirements for meat and meat products have been driven by market composition and consumption habits across countries. For instance, packaging for Chinese markets need to consider issues around domestic distribution, retail display, wholesale, and household storage; whereas for NZ, it is essential to extend the quality of chilled and frozen meat fabricated as primal, sub-primal and retail-ready forms for global distribution and long-term storage. Therefore, a successful meat packaging format must meet not only consumer expectations on product safety and quality, but also satisfy the needs for mechanical properties and economic impacts, such as durability, ease of machining and cost-effectiveness.

There are many packaging formats that are applied to chilled, frozen, and processed meat products to protect their safety, nutritional and sensory properties during distribution, storage, and display, as summarised in Table 1 [21,22,23,24,25,26,27]. These packaging formats can range from aerobic packaging for short-term retail display of chilled and frozen and thawed meat (e.g., overwrap), to those comprising materials with excellent gas barrier properties for longer-term storage and display of chilled, processed or cooked meat (e.g., vacuum packaging and modified atmosphere packaging).

Different packaging technologies have their own advantages and disadvantages in maintaining the quality and consumer acceptance of fresh and processed meat products. The oxygen-permeable films used for aerobic packaging allow oxygen to diffuse from the environment into the meat surface to encourage the blooming of the meat. This process involves the formation of oxymyoglobin, which is associated with the fresh bright-red meat colour preferred by consumers. However, the exposure to an oxygen-rich environment also accelerates the deterioration of meat quality due to the growth of spoilage microorganisms and severe oxidative damage of lipids and proteins. As a result, vacuum packaging was developed to extend the shelf-life of meat products by creating an anaerobic environment, which is generated when a vacuum is applied within the package to remove ambient air around meat before sealing the pouches or rollstock [5]. The vacuum packaging technique has been widely used in the meat industry for its ability to retard the growth of aerobic microorganisms and reduce the oxidative damage of proteins and lipids [28]. The trade-off is the production of deoxymyoglobin on meat surface resulting in a purple colour which has a lower rate of acceptability by consumers. This has led to the adoption of modified atmosphere packaging (MAP), which was optimised to improve the shelf-life and preserve the natural colour of chilled meat. Unlike vacuum packaging, MAP replaces the ambient air around the meat products with a gas mixture tailored to minimise deterioration while maintaining desired colour properties. In general, oxygen is blended with other gases such as nitrogen, carbon dioxide and carbon monoxide at different concentrations to create the ideal environment for optimum meat quality and shelf-life [29]. In this system, oxygen also promotes the formation of oxymyoglobin, keeping the bright-red colour of fresh meat, while carbon dioxide can inhibit the growth of microorganisms and extend the shelf-life. To prolong the bright-red colour of fresh meat, carbon monoxide has also been used, which produces carboxymyoglobin, which is more stable than oxymyoglobin [30]. Lastly, nitrogen is used as a filling gas to prevent package collapse and has no negative impact on the quality of fresh meat.

These different packaging formats are also commonly used together by the meat industry to create a variety of permutations, which optimise meat quality throughout its storage life. For wholesale and export markets, chilled meat is generally fabricated into primal or sub-primal cuts and packaged within flexible vacuum packaging or MAP, and then packaged within master packs at the meat processing facility. These master packs can also be vacuum or MAP for further protecting the safety and quality attributes during distribution. Similarly, frozen meat is packaged in shrink or vacuum packaging (primal and sub-primal) or high-abuse bags. However, some wholesale meat packaging formats are also used in retail.

At the retail level, the majority of chilled meat is prepared into retail-cuts and packaged in plastic trays with a transparent overwrap film or using MAP, with a small volume of aged meat products being available in vacuum packaged formats. Most large retailers (supermarkets) also have a frozen section for larger meat cuts and frozen and thawed section for retail cuts which are packaged using similar formats to their equivalent chilled products. In general, retail and portioned chilled meat prepared by an in-store butcher is packaged in oxygen-permeable packaging to ensure the bright-red fresh meat colour develops well during display for purchase by consumers. Whereas case-ready products prepared by the meat processor are packaged using vacuum, vacuum skin or MAP, which have a range of different gas-barrier properties (e.g., anoxic or aerobic environments) depending on the type of film used, and have been selected based on the desired appearance of the packaged meat [5].

## 3. Emerging Trends in AP Applications

**Figure 1:**
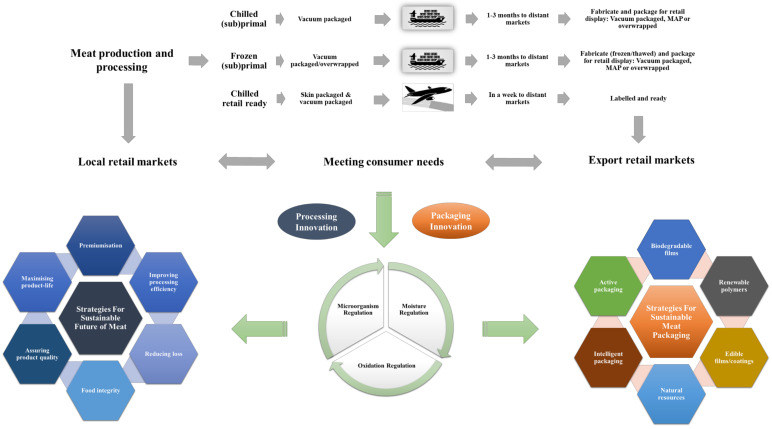
Example of sustainable strategies for meat processing and packaging innovations.

In recent years, the meat industry has faced challenges from the increasing consumer demand for non-animal protein alternatives in an effort to address the environmental and health concerns associated with meat production and red meat consumption [31]. As such, the value proposition of packaged meat needs to be reconsidered in response to these emerging changes in consumer preferences. Providing meat products with consistent and exceptional eating quality becomes an important strategy for the meat industry to meet consumer expectations [32]. Research in meat packaging has been recently focussed on developing more sustainable and fully biodegradable innovative packaging materials and functional packaging systems for the meat industry. Limited research however has been conducted about the role that packaging might play in keeping the quality of value-added products and improving processing efficiency (i.e., obtaining similar/improved quality with a shorter turnaround) within the processing plant. Research in meat packaging has focused on both materials (e.g., composite materials, sustainable polymers and multi-layer films) and functionality (e.g., active agents and intelligent features) for assuring product integrity [33]. In the short-term it can be expected that more environmentally-aware consumers will lead scientists and manufacturers to develop and use more sustainable meat packaging options to improve shelf-life as well as processing efficiency (Figure 1). The benefits of AP functionalities, such as antimicrobial, antioxidant and moisture regulation activities can be extended beyond preservation of meat quality during storage to other benefits such as meat processing optimisation and the creation of added-value products. This section discusses current and potential applications of AP to pre-rigor (hot) meat, wet- and dry-aged meat and frozen and thawed meat.

### 3.1. AP for Pre-Rigor (Hot) Meat

Pre-rigor meat, also called hot meat, is produced by fabricating a carcase immediately after slaughter, and the muscle has not entered rigor mortis when they are distributed to the market and purchased by consumers. Pre-rigor meat is well-known for its superior functionalities for producing processed meat, such as higher water holding capacity, binding and emulsifying properties and extractability of myofibrillar proteins [34]. Good meat quality has also been linked with pre-rigor meat such as tenderness and colour [35]. Pre-rigor meat is still physiologically active (i.e., high pH and available glycogen) when cooking, which results in distinctive flavour profile compared to post-rigor and aged meat due to the differences in the composition of flavour precursors (i.e., free amino acids, peptides and nucleotides) and key aroma compounds [36,37]. The flavour produced from pre-rigor meat is perceived as “fresh” and preferred by Chinese consumers as discussed in Section 2.1.2.

Extensive studies have been performed focusing on packaging materials for post-rigor and aged meat products, however, research on pre-rigor meat is scarce. The packaging formats such as wrapping and vacuum packaging for hot-boned meat could be applied to package pre-rigor meat. There is a range of natural antimicrobials being used for the preservation of food products, which may have potential to be used in the future-developed APs for pre-rigor meat. These antimicrobials include bio-preservative bacteria, naturally occurring organic acids, proteins and peptides, enzymes (e.g., lysozyme), and bacteriocins (ribosomal antibacterial peptides produced by bacteria), and some metallic nanoparticles (e.g., silver, copper, and gold).

Bio-preservative bacteria have demonstrated promising roles in the improvement of product shelf-life by altering the ultimate microbial profile on the meat surface with generally recognised as safe (GRAS) bacterial species which have limited or no spoilage potential. Lactic acid bacteria (LAB), particularly *Lactobacillus* spp., *Aerococcus* spp., *Carnobacterium* spp., *C. divergens* and *C. maltaromaticum*, are considered as potential bio-preservative agents since they are naturally found in vacuum packaged meat and they are able to survive extreme changes in conditions, for instance, multiple freezing/thawing and high-pressure cycles [38]. In addition, they may be able to produce a range of antibacterial metabolites, such as bacteriocins, lactic, acetic and propionic acids, carbon dioxide, hydrogen peroxide and anti-fungal peptides, which enable them to successfully outgrow other spoilage bacteria and achieve effective bio-preservation.

Bacteriocins are antimicrobial peptides that can be produced by some naturally occurring bacteria from refrigerated meat, such as LAB and *Enterobacteriaceae* (*Serratia* spp.) which can retard or inhibit the growth of other bacteria [39]. They are thought to exhibit biocidal activity owing to the positively charged groups on the bacteriocin peptide interacting with negatively charged phosphate groups on the surface of the cell membrane in order to bind to the bacterial cell, allowing the hydrophobic groups on the bacteriocin peptide to penetrate into the cell membrane, creating pores which ultimately result in cell death [40]. Nisin and pediocin are the most commonly explored bacteriocins for antimicrobial packaging of meat and other muscle foods such as poultry and seafood [41]. As bacteriocins are most effective against Gram-positive bacteria and possess a narrow antimicrobial spectrum, they are typically used alongside other antimicrobials [42].

Naturally occurring organic acids such as formic, acetic, propionic, butyric, lauric, lactic, sorbic and benzoic acids (and their salts) exhibit broad antimicrobial activity against yeasts, moulds and bacteria [43]. Acetic and propionic acid were shown to be most effective in inhibiting the growth of *L. sakei* and *S. liquefaciens*, as well as *Enterobacteriacae* and LAB on bologna and pastrami when stored at refrigerated temperatures for up to 21 days [44]. Antibacterial agents from plant sources such as herbs (e.g., garlic, oregano, cinnamon, clove, rosemary and thyme, etc.) and other medicinal materials like flowers, buds, roots and leaves have shown the potential to be incorporated into AP of meat as crude extracts or essential oils. Some examples of antimicrobials from plant extracts are summarised in Table 2. The effectiveness of essential oils against meat-borne bacteria depends on the pH, temperature, and level of microbial contamination of meat products. A high concentration of essential oil is generally required to achieve desired antibacterial properties, thereby its application on the meat could be limited by the intense aroma of essential oils which may negatively affect the organoleptic quality of meat [45]. Combining a low concentration of these plant extracts with other antibacterial agents (e.g., nisin) or applying novel technologies such as encapsulation of essential oils could reduce the organoleptic impacts of essential oils [26]. Similarly, some minerals including metal ions (e.g., silver, copper, iron and zinc) and metal oxide nanoparticles (e.g., zinc oxide, titanium dioxide) are well known for their antimicrobial properties, and are generally incorporated into polymers and used for regulating the proliferation of microorganisms during storage [46]. However, the impact of these minerals and nanoparticles on the material properties of packaging films and packaging techniques used for the different types of meat products, and the potential impacts on human health due to migration into the food or wider environment need to be carefully considered.

### 3.2. AP for Post-Rigor (Aged) Meat

Post-mortem ageing is a widely applied practice by the meat industry to achieve premium quality of meat. Consumers currently have an increased understanding that well-aged meat can provide eating quality resulting from satisfactory tenderness and a characteristic aged flavour. There are generally two types of ageing techniques: wet-ageing and dry-ageing. While meat ageing can add value to the product, ageing techniques do have challenges for meat processors, including high energy use, long processing times and potential losses. This section reviews some of the current challenges associated with wet- and dry-ageing to understand how recent advancements in AP could contribute to optimise the ageing process.

#### 3.2.1. Wet-Aged Meat

Most primal meat cuts are vacuum packaged/wet-aged in commercial practice, especially during shipping and storage, before they are fabricated into retail cuts. Wet-ageing is considered as the most practical method of ageing in the meat industry owing to ease and flexibility for storage and transport, and for having very low losses during the ageing process [25]. Therefore, wet-ageing is applied in meat industry as a storage strategy to extend the shelf-life of fresh meat during domestic and long-distance global distribution [1]. Vacuum packaging of fresh meat at a low storage temperature is recommended to maximise the shelf-life and ensure the premium quality of chilled products. An ultra-low chilling temperature of −1.5 °C has been routinely applied for chilled meat distributed from NZ to international markets like China, Malaysia, and the US. A product shelf-life of around 12 weeks for lamb [1] and 20 weeks for beef [47] can be achieved with high standard process hygiene and careful temperature control during storage and transport. The trade-off, however, is the energy and operational costs for running at a freezing temperature throughout the storage and cold chain distribution, which also creates significant environmental impacts.

Using a higher ageing temperature favours the enzymatic activities to improve the ageing efficiency, and also reduces the time and energy needed for producing well-aged meat products. Wet-ageing at higher temperatures (e.g., 3–8 °C) has been used to accelerate the ageing process for developing desired tenderness and flavour of meat, however, the product life and quality of aged products is compromised as a result of quality deterioration from the growth of toxin-producing pathogens (e.g., non-proteolytic *Clostridium botulinum* type B) [48], spoilage microorganisms [1] and increased drip loss. One of the most detrimental impacts of ageing is the development of drip, also known as exudate, as a result of proteolytic activity. Drip is a red, concentrated solution of primarily intracellular proteins, including myoglobin and glycolytic enzymes. The generation of drip during ageing/storage could have impacts on meat quality in several ways. Firstly, excessive drip can reduce meat functionality and quality, which is commonly rejected by consumers [49]. As drip generally contains mainly water-soluble protein fractions including enzymes, proteins, peptides, amino acids and nucleotides, increased drip loss could also impact the nutritive value of meat [50]. Further, drip can also serve as an excellent substrate for the proliferation of spoilage microorganisms and toxin producing pathogens.

Advancement in packaging research may identify opportunities to apply AP materials with greater moisture regulation abilities, which may reduce the direct contact of exudates with meat and minimise deterioration. Packaging formats which incorporate a moisture absorber could provide effective control of drip accumulation inside the packaging, thereby suppressing the growth of microorganisms [7]. Absorbent pads are the most used moisture regulation system in the current industry setting, consisting of a super absorbent polymer and covered with layers of microporous/non-woven polymer [8]. This approach is typically seen in overwrapped and MAP fresh meat in retail and is sometimes used in vacuum-packaging of large meat cuts (e.g., beef striploin and lamb leg). This type of approach can be improved further through the incorporation of active agents such as antimicrobials, antioxidants, oxygen scavengers and/or pH control agents, to achieve multiple functions in a single system [10,51]. There have also been a few studies which have reported the use of absorbent pads containing nanoparticles or essential oils [52] to control the microbial spoilage of fresh meat in tray-formatted packaging, however research on their application in vacuum packaged meat is scarce. Future research on the application of multifunctional absorbent pad systems for improving the quality and product life of vacuum-packaged/wet-aged meat products is in need.

#### 3.2.2. Dry-Aged Meat

Dry-aged meat products have been recognised as a premium food sought after by consumers for their characteristic dry-aged meat flavours such as nutty, roasted, sweet and umami [25]. However, dry-ageing is not commonly applied by the meat industry due to concerns over excessive product losses resulting from ageing and trimming, as well as oxidation and microbial contamination which may lead to a low product yield, high retail price and risk of quality deterioration [25]. Further, the premium quality of dry-aged meat requires a long ageing time (over 4–6 weeks) inside an ageing chamber with critical control of high standard hygiene practices and ageing parameters (temperature, humidity, and air velocities). These practices can therefore generate extensive costs in energy and space.

One recent advancement in dry-ageing packaging demonstrated the feasibility of dry-ageing meat in a water-permeable ageing bag, known as ‘in-bag dry-ageing’, for producing a premium dry-aged product with more consistent quality and microbial safety [53,54]. The main function of the ageing bag is to allow moisture to be released from the meat while also preventing the direct contact of the meat with the ageing environment, reducing the risk of contamination and excessive oxidation. In fact, the use of dry-ageing bags could reduce the need for a critically controlled ageing environment since the rate of moisture release is mainly regulated by the moisture permeability of the ageing bag [53,55]. Further, recent studies by Zhang, Yoo and Farouk [53] and Zhang, et al. [56] found that the ageing chamber air velocity had no major impact on the quality and biochemical properties of in-bag dry-aged beef. Therefore, in-bag dry-ageing shows high potential for producing sustainable and high-value meat products in the future. Innovations in packaging materials for in-bag dry-ageing applications should focus on improving the efficiency of dry-ageing through reducing energy requirements and trim volumes.

Moisture evaporation during dry-ageing has been suggested to contribute to the development of the characteristic dry-aged flavour through its concentration effect on the flavour compounds [57]. Therefore, accelerating the development of dry-aged flavours could be achieved through the manipulation of ageing temperature and water permeability of the ageing bag. Several types of ageing bags have been reported in the literature which mainly differ in the water vapour transmission rate and polymer types [54,55,58,59]. The most commonly used TUBLIN^®^ plastic bag is a thermoplastic elastomer made of flexible polymer and rigid polyamide with high permeability to water vapour, allowing rapid and even evaporation of water from meat, while also limiting the access of oxygen and microorganisms to the meat [53]. The resultant aged meat products have consistent quality and lower product waste than a traditional dry-ageing approach [54,60], with major implications for the environmental footprint of the product. Another strategy to accelerate the development of desired dry-aged quality is to increase ageing temperatures (e.g., 5–10 °C), which improve the enzymatic degradation of lipid and protein by proteases, peptidases and aminopeptidases, and result in the release of free amino acids, fatty acids and short peptides, that may contribute directly to flavour [61]. Further, the use of a higher ageing temperature may also increase the moisture evaporation rate, resulting in the dry-aged products reaching a desired quality within a much shorter ageing time (1–2 weeks). As a result, the product would have a lower demand for refrigeration and storage space compared to traditional dry-ageing regimes at lower temperatures (1–4 °C) for a longer ageing time (4–6 weeks). However, a higher ageing temperature may also promote the growth of spoilage microorganisms including moulds and yeast. Developing innovative packaging materials with antimicrobial or microorganism regulation functions could have potentials to be applied for dry-ageing of meat to improve the processing efficiency.

So far, there is only one study by Gudjónsdóttir, et al. [62] which has applied an antimicrobial packaging film for dry-ageing, prepared from electro-spun chitosan fibre. The chitosan treatment showed reduced muscle denaturation during ageing, reduced microbial counts (included yeasts and moulds), improved product yield and also gave rise to a lighter appearance compared to the traditional non-bag dry-ageing treatment. The study demonstrated the potential of using electro-spun chitosan fibre as a wrapping material for improving the quality of dry-aged meat. However, this type of packaging may be impractical due to the slow and costly production process and the weak nanomembrane strength. Future studies are warranted to determine the impact of such packaging material and other antimicrobials as described in Section 3.1 in improving the efficiency of dry-aged flavour development across a wider range of temperatures and air velocities.

### 3.3. AP for Frozen and Thawed Meat

During frozen storage, meat quality deterioration which results from enzymatic, physical, biochemical and microbiological activities is retarded due to the low temperature and the absence of free water in meat extending shelf-life. Freezing plays an essential role in the meat export industry by assuring the safety and superior quality of the meat products being supplied overseas, particularly for distant markets, for instance, the export from NZ to China, the US and European countries. However, the recent COVID-19 pandemic has caused severe disruptions within the meat supply chain resulting in the temperatory closure of processing plants and disruption to trade routes [63]. In many countries, the “stay at home” measures have led to sudden changes in consumer purchasing behaviour, and frozen products have become popular for their extra long shelf-life [64]. As a widely accepted technology for preservation, freezing of meat carries the advantages that consumers can choose the consumption date after long and variable periods of time, without significant changes in meat quality compared to fresh meat. Most of the meat exported from NZ is still shipped frozen (Section 2.1.1), therefore there is a need to consider strategies to improve the quality of frozen and thawed meat products.

Lipid and protein oxidations are the main causes of quality deterioration during frozen storage and the subsequent thawing process, which lead to discolouration, texture changes and the formation of off-flavour and toxic compounds [65]. Some strategies have been researched to improve the oxidative stability of meat products during frozen storage. For instance, the use of ultra-low (<−20 °C) and ultra-fast freezing techniques have been suggested to extend the product shelf-life [66], whereas the economic and environmental impacts may limit industrial applications due to the additional capital costs for the freezing unit and its energy consumption. Frozen storage at higher temperatures (e.g., −12 °C) could be possible, and may result in comparable quality to meat stored at −18 °C [67]. However, if the product shelf-life is compromised a long-term storage may not be feasible.

Another strategy which has been commonly used in meat industry is to reduce the oxygen content in the packaging through vacuum packaging or MAP (as described in Section 2.2). Although using vacuum packaging or MAP combined with a good oxygen barrier will limit oxygen in the food packaging, these techniques do not completely remove the oxygen inside the packaging, with a remaining residual oxygen-concentration between 0.5% and 5% [10]. However, lipid oxidation could be triggered even at low oxygen conditions around 0.05% [5]. The application of antioxidant packaging may become the emerging strategy for improving the quality of meat products during frozen storage and following thawing and storage at retail. Antioxidant AP has been suggested to improve the quality and safety of fresh and processed meat [65]. Both synthetic and natural antioxidants may inhibit lipid and protein oxidation, retard the formation of off-flavours and odours, as well as maintain colour stability in meat [7]. The application of oxygen scavengers is one of the most widely used commercial AP technologies aiming to remove residual oxygen [68]. Iron and ferrous oxide-based oxygen scavengers (provided in packet/sachets) are the most effective and commonly used scavengers, which remove residual oxygen in pack through its reaction with iron. However, most oxygen-scavenging systems need several days to remove oxygen present in the initial headspace which may be too slow for thawed meat products, which can deteriorate faster than fresh products. To inhibit metallic tastes from the scavenger being imparted to the food, non-metallic oxygen scavengers such as ascorbic acid, sulfites, catechol, ascorbate salts, and enzymes like ethanol oxidase have also been used for inhibiting oxidation [68]. For a wide range of food applications, the performance of ascorbic acid and ascorbates-based oxygen scavengers is considered sufficient and are commercially available (e.g., Daraform^®^ and Freshilizer^TM^). Likewise, the incorporation of ascorbic acid into high barrier bio-polymer films or containers has also been explored to protect meat products from oxidation [10].

A recent trend in antioxidant AP research for meat is the use of natural antioxidants instead of the use of synthetic additives. The most common natural antioxidants are α-tocopherols, polyphenols (e.g., quercetin, catechin, flavonoids), essential oils from spices and grain residues (e.g., cinnamon, lemongrass, clove, thyme, ginger, oregano) and plant extracts (e.g., rosemary, grape seed, green tea, oregano, and pomegranate peel) [69,70]. The use of natural plant extracts in AP for controlling oxidative changes in meat products during storage and extending shelf-life has become a popular research topic, as it is suspected to work well without compromising the sensorial quality of meat [65]. For example, recently published studies have applied *Cucumis metuliferus* fruit extract loaded into cellulose acetate coatings for preventing lipid oxidation in fatty food [71], pomegranate peel extract encapsulated within chitosan/poly (ethylene oxide) nanofibres for meat preservation [72] and grape seed extract directly incorporated into chitosan film and used in the packaging of chicken breast under 4 °C for 20 days [73]. Natural extracts which are rich in phenolic compounds and essential oils also show good antioxidant activity when incorporated into film materials [74]. Research into natural extracts and essential oils and their application to meat packaging systems has revealed their potential to counteract the detrimental effects of high oxygen concentrations in packaging on the oxidative stability of fresh meat. Further, some essential oils and plant extracts also have antimicrobial activities (Table 2). For instance, essential oils of oregano [75] and thyme [76], and seaweed extracts [77], which could become a promising applications for multifunctional AP. However, studies of their applications on frozen and thawed meat products are scarce.

**Table 2:** Progress in active packaging with antimicrobial and antioxidant functions and their meat applications.

Active Components	Matrix	Meat	Target Microorganism	Main Effects	Reference
***Antimicrobial packaging***					
Tea tree oil	Chitosan electrospun nanofilms loaded with tea tree oil liposomes	Chicken	*Salmonella Enteritidis and Salmonella Typhimurium*	The nanofibers membrane inhibited 99.99% *Salmonella* in chicken after 4 days treatment without an impact on the sensory quality.	[78]
Thyme EO	Silk fibroin nanofibers	Poultry	*Salmonella Typhimurium*	Nanofibers decreased the count of *Salmonella Typhimurium* from 6.64 to 2.24 Log CFU/g.	[75]
Oregano EO	Sodium alginate film	Ham slices	*Listeria monocytogenes*	The film caused approximately 1.5 log reduction in *Listeria* population at 8 and 12 °C at the end of the storage period, and almost 2.5 log reduction at 4 °C.	[76]
Gallic acid + chitosan or carvacrol + chitosan	Starch	Ham	*Carnobacterium Leuconostoc Brochothrix Listeria monocytogenes*	Starch films with chitosan and carvacrol fully inhibited *L. monocytogenes* growth throughout 4 weeks of storage, starch films loaded with chitosan or chitosan and carvacrol delayed growth of ham microbiota by 1–2 weeks.	[79]
***Antioxidant packaging***					
Rosemary extract	Low density polyethylene	Pork Patties	-	Significant inhibition of lipid oxidation.	[80]
Chitosan	Gelatin film	Beef fillet	-	Lipid oxidation was slowed by chitosan in concentration-dependent manner; reduction of the formation of metmyoglobin.	[81]
Palladium (Pd) (+ hydrogen)	PET/SiOx/Pd	Cooked cured ham slice	-	Prevention of discoloration (redness).	[82]
Cinnamon (85%) + Rosemary (15%) essential oil	Whey protein	Pork salami	-	Significant inhibition of lipid oxidation.	[83]
Green tea extract	Polyamide	Minced beef	-	The film had excellent antioxidant capacities and increased the shelf life from 6 to 23 days.	[84]
***Antioxidant + Antimicrobial packaging***					
Postbiotics of *Lactobacillus plantarum*	Bacterial nanocellulose	Ground beef	*Listeria monocytogenes*	The film caused a reduction (~5 log cycles) of *L. monocytogenes* counts in ground meat. The postbiotics of *L. plantarum* revealed a moderated antioxidant activity in meat.	[85]
*Anethum graveolens* EO	*Plantago major* seed mucilage coating	Fresh beef	*E. coli, Staphylococcus aureus; and fungi*	Extended the shelf-life of meat from 6 to 18 days and inhibited bacterial growth and slowed down the oxidative changes	[86]
Catechin and lysozyme	Gelatin film	Minced pork	TVC, yeast and mould	Extended shelf life and lowered the total plate count, yeast, and mould. Successful inhibition of lipid oxidation and microbial growth.	[87]
Clove and cinnamon	Corn starch	Beef fillet	*Pseudomonas* spp., *Enterobacteriaceae*, LAB	Reduction in microbial populations, improved meat colour stability at the end of storage.	[88]
*Origanum virens* EO	Whey protein coatings	Portuguese sausage	TVC	Inhibition of the total microbial load, higher acidity and protection against discolouration.	[89]
*Terminalia arjuna* extract	Maltodextrin and calcium alginate	Chevon sausages	TVC, yeast and mould	Lipid oxidation was inhibited, and yeast and mould counts were lowered.	[90]
Ethanolic propolis extract	Chitosan film enriched with cellulose nanoparticle	Minced beef	*Pseudomonas spp*., LAB, and *Enterobacteriaceae*	Microbial growth was delayed, lipid and protein oxidation were retarded.	[91]
Resveratrol	Gelatin/zein fibre mats	Fresh pork	*E. coli*, *Staphylococcus aureus*; TVC	Good antibacterial activity against *E. coli* and *S. aureus*, antioxidant activity to inhibit discoloration and extended shelf-life.	[92]
Pomegranate peel extract	Chitosan/PEO nanofiber	Fresh beef	*E.coli* O157:H7	Extended the shelf-life without losing sensory properties; reduced *E.coli* O157:H7 up to 2.96 or 5.80 log CFU/g at 4 or 25 °C, respectively.	[72]

### 3.4. Polymers Used in Sustainable Packaging

Polymers used in packaging can generally be considered as non-edible or edible and are derived from renewable or non-renewable feedstocks. In addition, packaging polymers are generally also considered in view of their end-of-life disposal, whereby the polymers are either degradable or non-degradable. Naturally occurring polymers such as polysaccharides, proteins and lipids, as well as those derived from biosynthetic pathways (such as polylactic acid) are bio-degradable in general [11].

The most common packaging materials (Table 1) applied to meat products include polyamides (PA), polyethylene (PE) and polyvinylidene chloride (PVDC), and their monomers are derived from non-renewable resources (e.g., natural gas, crude oil and coal) followed by polymerisation to produce polymers with different material properties. Selective oxygen permeability may also be required for some case-ready MAP or retail packaged fresh meat, in these cases polyvinyl chloride (PVC) or low-density polyethylene (LDPE) are often used. Some low-cost polymers, such as polyethylene terephthalate (PET), polypropylene (PP) and PE, are also incorporated to develop cost-effective packaging [93]. However, these synthetic polymers are not biodegradable, and when combined into multi-layer films cannot be easily separated for recycling, and become a contaminant in recycling stream for mono-materials such as PET [94].

Growing demand for sustainable packaging materials has triggered a shift towards the development of renewable polymers with improved functional features [4,11]. Renewable polymers can be produced from bio-based resources or through biosynthetic pathways. Natural bio-polymers include polysaccharides, proteins and lipids from plant or animal origin. Similarly, natural polysaccharide and lignocellulosic feedstocks can also be used to produce biosynthetic polymers, such as polylactic acid (PLA) through fermentation processes, and are currently the most popular renewable polymers in use [95]. Nevertheless, most natural polymers have poor material properties such as poor gas and water barrier properties and thermal stability [96]. For example, PLA exhibits poor transverse strength, brittleness, and gas barrier properties [97] and polysaccharide-based packaging has weak moisture barrier properties and flexural rigidity. However, these limitations can be improved through chemical crosslinking or blending with other polymers [98].

Recent research into AP for meat products has extended into the potential for active components to be incorporated into edible films and coatings to address both environmental concerns, by utilising food co-products as a source of active compounds or polymers, and consumer demands for natural foods [99]. In general, edible packaging is used for cooked or ready-to-eat meat products and derived from edible bio-polymers produced as low-value co-products from food industry. Polysaccharides (e.g., chitosan, cellulose derivatives, alginate, starch) and proteins (e.g., collagen, zein, casein) are the most popular polymers demonstrating promising applications for edible packaging [100]. The packaging derived from bacterial polyhydroxyalkanoates, such as poly (3-hydroxybutyrate), poly (3-hydroxyvalerate), and their copolymer (Biopol^®^) are also considered edible. They biodegrade more readily than polycaprolactone and PLA, being degraded within 3 months during soil burial or composting [101]. Further, composite films and coatings can be prepared from mixtures of protein and polysaccharides. However, most plastics derived from natural polymers are often much more expensive than the current plastic packaging (e.g., $5–$10/kg vs. $0.05–$0.30/kg) [102]. Food-grade natural polymers are likely to be more expensive due to the implementation of additional hygiene measures. Thus, the added costs to manufacture edible packaging could lead to a higher sale price which may hinder the adoption by the industry [99]. There is a continued trend for incorporating active compounds into edible packaging [73] with tailored functionalities for ensuring product quality.

## 4. Manufacturing Techniques and Policies for AP

### 4.1. Manufacturing of Packaging Films

Plastic packaging for meat products is generally considered in terms of its physical properties including rigid, semi-rigid and flexible. Rigid trays are pre-made by packaging manufacturers prior to their use in the meat processing plant, butchery, or supermarket by compression moulding, which involves shaping a rigid sheet or film to the shape of a mould using heat and pressure to form trays. The process is more cost-effective than using injection moulding due to the lower cost to produce the mould (only one side of a mould is required) and its higher throughput. Rigid trays can be produced using either a mono-layer (mono-material) film, or a multi-layer film if specific barrier properties are required. These rigid packages are primarily used for individual packaging of portioned meat products in supermarkets, then covered with overwrap or sealed with film for retail display.

Flexible and semi-rigid packaging such as chub, pouches or thermoformed webs are produced in the meat processing plant, butchery, or supermarket from rollstock or film manufactured by packaging companies. Flexible packaging films used as overwrap, chub or pouches can be produced from mono-materials such as LDPE or PVDC. In these cases, film or rollstock is produced by extruding the mono-material and then shaping it into a film using either a flat filming technique (where the material is forced through a die with a narrow slit at the end of the extruder, also known as cast film extrusion) to produce a sheet, or a film blowing technique (where the material is forced through a cylindrical die and expanded into a blown tube shape by air) to produce a tube, or cut to produce two sheets. However, some packaging formats (e.g., vacuum) utilise multi-layer films to improve overall packaging performance. In addition, most of the semi-rigid packaging materials (e.g., thermoformed) also utilise multi-layer structures, produced analogously to flexible multi-layer films.

For most applications, mono-material films are unable to meet all the packaging requirements for the distribution and sale of meat products thereby multi-layers packaging films with different types of polymers are commonly used (Table 1). Multi-layer films used in food packaging typically consist of 3 to 7 layers, however it is viable to produce up to 12 layers for use in other applications [28]. In general, the outermost layer is selected for abrasion resistance and printability, with middle layers selected for toughness and gas barrier properties, and innermost layers (next to the meat) selected for heat sealability and compatibility with the meat products. The conventional techniques used to manufacture multilayer films include coating, lamination, co-injection with stretch blow moulding and co-extrusion [103]. Besides, several advanced preparation techniques, such as surface modifications using UV-curing technology or atmospheric cold plasma treatment, nanostructured multilayer films based on the layer-by-layer (LBL) fabrication approach or electrohydrodynamic processing, have been applied to overcome the limitations of conventional preparation techniques [104].

### 4.2. Introduction of Active Agents

The technologies available to introduce active compounds to packaging can be used in-line with the processes used to manufacture sheets and films for rigid, semi-rigid and flexible packaging. Active agents can be incorporated into packaging through a variety of methods, but in general are included in a mobile state (allowing the active agent to migrate to the surface of the food) or in an immobilised state (preventing the active agent from migrating to the surface of the food) [13]. For incorporating active agents into packaging which are capable of migrating to the food surface, the most common methods include direct blending of actives to the polymers during film extrusion [105], direct coating or spraying onto the surface (the surface will be in contact with food), non-covalently immobilising by mixing with another polymer and coating on the film surface or addition through layer-by-layer deposition. The introduction method depends on the release mechanism and nature of the active substances which include mainly two types: controlled release by direct contact between the food and packaging material (non-volatile substances), or gas-phase diffusion (volatile substances) from the packaging layer to the food surface [79]. In many cases, the substances responsible for active function of the packaging are applied directly to the surface of the food product through its contact with the plastic film coating (of which the active compound may be either dispersed within the polymer film or concentrated at its surface as a coating). The active compounds may be encapsulated within a microencapsulated or electro-spun polymer to improve its action over time [12]. By contrast, active agents which must be prevented from migrating to the food are added to packaging film and immobilised through covalent bonding [106] or polymeric grafting (e.g., photo-grafting).

Indirect headspace diffusion method results in a high degree of migration of the actives onto the meat through sorption from the headspace onto the meat surface but limits its efficacy to the vicinity of its exposure. By contrast, direct contact methods tend to have greater immediate efficacy, as the active acts on the entire product surface that the packaging is in contact with, which in the case of fresh and processed meat is sufficient as the interior may often be considered sterile, and most of the deterioration occurs at the surface where microorganisms are growing [107]. However, it can cause contamination of food as not all active agents are edible. Direct contact between the AP film and the food surface may lead to changes in quality parameters such as colour or taste, thus affecting organoleptic acceptance of the product and causing regulatory concerns [2]. Further, the immediate, direct contact between the active compound and food surface may result in the active compound being rapidly depleted in a chemical reaction, whereby protection of the food ceases, and the quality of the food degrades more quickly [108]. This type of depletion is seen when nisin is in contact with meat, and becomes inactivated through an enzyme-mediated reaction with glutathione, highlighting the importance of encapsulation techniques for retaining activity over time.

### 4.3. Legislations for Food Contact Materials in China and NZ

Food safety is a global priority and is one of the major objectives of the current food legislation in order to promote sustainable development of meat industry. Packaging-related laws and regulations are being promulgated and implemented globally, which play an important role in legal protection for product safety whereas also constrain the application of AP in food system. National regulations concerning Food Contact Materials (FCM) have been enacted worldwide, but they vary between countries and not all of them refer to AP specifically. Furthermore, when it comes to nanomaterials used within AP, the safety evaluation and approval process for the use of some nanoparticles (particularly metal ions) in food packaging remains challenging as there are difficulties in the evaluation of their safety and constraints associated with their assessment in the current policies. Therefore, to scale up and industrialize the AP technologies, legislative and regulatory issues must be addressed.

FCM used in China are regulated by the Food Safety Law of the People’s Republic of China 2015. There are over 130 National Standards and 125 Industrial Standards for food packaging materials and containers in China which set specific safety requirements (evaporation residue, migration of hazardous, etc.) for FCM including material groups (e.g., paper products, metal products, ceramics), specific products and food contact additives. New regulations for FCM were introduced in 2017, of which requirements and standards regarding general safety, and the scope and restriction of various additives for producing FCM and products were included. All FCM and food contact articles made available in China must be compliant, properly labelled with the product name and material and provide a declaration of compliance according to the requirements of National Standards. New regulations which cover paper and paperboard for food contact use are more adequate and flexible, and provide a new basis for the general requirements and specific safety and marketing issues related to active and intelligent packaging in China. Besides, when manufacturing new materials and additives in food packaging materials (including resins and additives), they must pass the safety assessment of the National Health Commission of the People’s Republic of China and obtain the registration approval, the manufacturers then ensure the FCM complies with the food safety standards, conducts migration testing to verify compliance and provides product information to downstream users including the declaration of compliance and labelling [109].

NZ regulates FCM through the binational agency Food Standards Australia NZ (FSANZ) under the joint Australia NZ Food Standards Code, enforced by the Ministry for Primary Industries. Under the code, FCM are regulated based on the intended function of the substance in three categories: food additive, processing aid and package. Under the regulation by FSANZ, the food packaging must be safe, and its contact with food should not result in the food exceeding the limit set for the level of toxicants in food. For instance, Standard 1.4.1 describes the contaminants and natural toxicants in food and establishes maximum levels of metal, non-metal and natural toxicants that are allowed in food (detailed in Schedule 19 of the Code) [110]. FSANZ recently reviewed the existing regulatory framework to determine its adequacy for managing the risk of migration of chemicals from packaging to food. It was determined that chemical migration from packaging to food posed minimal public health and safety risk and the proposal to regulate food packaging was abandoned in favour of non-regulatory guidelines on food packaging [111].

## 5. Challenges and Future Perspectives

Extensive research has been carried out in developing AP as novel packaging solutions in the food industry whereas their commercialisation and adoption in meat industry is scarce. To the best of our knowledge, both China and NZ food industries are interesting in incorporating AP into their products while the widely uptake of this technology remains to be seen. One example of AP application in horticulture industry in NZ is to include an ethylene (a ripening hormone) regulator to absorb or release ethylene in the packaging of fresh fruit, which can preserve its freshness through using ethylene absorber sachets or control the ripening process using ethylene release capsule. Coating prepared from chitosan, starch or gelatine has been used in China for fruit preservation (e.g., antimicrobial and delaying spoilage). The major hurdles for the commercialisation of AP are related to the need for more evidence to show its effectiveness in-practice, perceived adverse effects on product quality, challenges in assuring their safety, and the challenges of producing AP on an industrial scale, including technology transfer issues, the cost of large-scale preparation or production, and rigorous legal and regulatory constraints. There are also concerns that using pouches or sachets may introduce new substances into the food as a result of migration, resulting in interactions between active agents and other packaging materials, or that active substances or their degradation and/or reaction products may migrate from packaging materials into the food, negatively impacting its toxicological properties. Consumers have also indicated that there is a perceived risk to health and safety with the introduction of nanotechnology, especially the antimicrobial substances like silver nanoparticles or ionic doped glass microparticles, which are generally toxic at low concentrations.

Further, the use of some active compounds is still controversial. For instance, antioxidants like butylated hydroxytoluene (BHT, E321) and butylated hydroxyanisole (BHA, E320), which are GRAS compounds and permitted to be use at the concentrations observed in food preservation. In addition, some of the currently used active agents show limited efficacy against microorganisms, lipid oxidation, low thermal stability and mostly function via direct contact. Additionally, it remains a challenge to develop active materials that can maintain their original mechanical and barrier properties after addition of the active components. Finally, one major obstacle is that certain AP formats which show a high activity on in vitro systems do not present this activity when they are tested in meat. Therefore, the efficacy of the specialised packaging in the meat industry deserves further investigation and clarification.

The future advancements of applying AP in meat industry could consider developing packaging materials with multifunctional approaches which incorporate different actives or a compound with several bioactive functions, to take advantages of their differing modes of action and efficacies over time. Such applications are especially relevant for processing and preservation of meat products, since the quality decay generally links with complex reactions between physical, biochemical and microbial activities. Further, the growing demands from consumers for high-quality convenient food (e.g., ready to cook/heat/eat) and flexitarian diet could impose the need to understand the interactions of different food components from plant and/or animal origins, and also lay a new ground for novel application of AP. Another emerging area of interest could be the application of AP for centralised packaging system of retail-ready meat products (e.g., skin packaging). This type of packaging approach may allow meat products to be shipped to the distant retail markets (e.g., from NZ to China) in a master package or distributed directly to the consumers who place the purchase through e-commerce.

## 6. Conclusions

Understanding consumer consumption habits, market composition and distribution channels for meat in China and NZ can assist with the customised design of packaging for improving processing efficiency, product quality and extending shelf-life of meat. AP is a promising technology for supporting the sustainability of the meat industry through improved packaging stewardship and the shelf-life of meat products. AP with antimicrobial activity may be utilised to extend shelf-life of pre-rigor meat through incorporation of natural antimicrobials into packaging formats like wrapping and vacuum-packaging. Further application of antimicrobial AP to tailor dry-ageing process through regulating the growth of microorganisms to facilitate the development of unique flavour profile and extend shelf-life. The moisture regulation system of AP combined with antimicrobial functions may have promising potentials for producing wet-aged meat with improved quality. Quality decay due to freezing and thawing of meat could also be improved using AP of antioxidant and antimicrobial functions. Continued innovation in both packaging materials and active compounds will be needed for AP applications in the meat industry in order to meet both technical and regulatory requirements for adoption.

## Figures and Tables

**Table 1:** Summary of packaging formats used for wholesale and retail meat products.

Packaging Format	Meat Type	Description	Key Polymer Requirements	Example of Packaging Materials	Key Benefits	Potential Trade-Offs
***Wholesale***						
Individually wrapped	Primal cuts	Meat cuts are individually wrapped in materials such as a sheet, stock netting or bag.	- Physical barrier - Good toughness, puncture resistance, gas, moisture and grease barrier properties in multilayer films	- Carton liners: LLDPE - Stockinette bag: cotton, LDPE, HDPE - Stock netting: PET, natural rubber - Shrink multilayer films: ULDPE/EVA/PVDC/EVA/ULDPE, m-LLDPE/EVA/PVDC/EVA/m-LLDPE, m-LLDPE/LLDPE/Tie/EVOH/PA, ION/LLDPE/Tie/EVOH/PA, ION/EVA/LLDPE/PVDC/o-PA	- Low cost - Low technology - Amenable to VP	- Wastage as more plastic is used - Susceptible to oxidation and freezing damage
Layer packaged	Sub-primal (e.g., flank, backstraps)	Meat is packaged into a carton containing at least two layers of meat. The most commonly used to layer small cuts.	- Physical barrier	- Carton liners: LLDPE - Stockinette bag: cotton, LDPE, HDPE - Stock netting: PET, natural rubber	- Low cost - Low technology - Amenable to VP	- Difficult to separate individual cut when frozen - Susceptible to oxidation and freezing damage
Multi wrapped	Primal cut (e.g., chuck tenders, lamb racks)	Meat is packaged into a single bag or covering, i.e., containing two or more cut items.	- Physical barrier	- Carton liners: LLDPE - Stockinette bag: cotton, LDPE, HDPE - Stock netting: PET, natural rubber	- Low cost - Low technology - Reduced volume - Amenable to VP	- Difficult to separate individual cut when frozen - Susceptible to oxidation and freezing damage
***Retail Ready***						
Tray overwrapped	Portioned meat; Cooked or processed meat	Meat is packaged onto a tray (rigid or expanded), typically containing a drip containing device and wrapped with a highly oxygen permeable film.	- Physical barrier - Oxygen permeable - Good clarity and gloss	- Tray: EPS, o-PP, PS, PET - Monolayer film: PVC, LDPE - Multilayer films: m-LLDPE/LLDP/Tie/LLDPE, m-LLDPE/LDPE//LDPE/Tie/PA/EVOH/PA - Soaker pad: cellulose, silica gel inside covering	- Low technology - Automatable for high throughput	- Short shelf-life due to oxidation and spoilage - Large pack volume
Clipped chub or vertical pouch	Ground meat (raw or cooked, e.g., sausage meat)	Packaging pumpable solids. Vacuum evacuation can be applied to the meat product by removing air around the filled chub film before sealing or clipping the remaining end of the film.	- Good toughness and puncture resistance - Oxygen barrier	- Monolayer films: PE, PVDC- Multilayer films: LLDPE/PVDC//PA	- Low cost - Lightweight - Reduced packaging - Shelf-life extended - Amenable to VP	- Limited to certain meat type - Low clarity - Possible leakage from clip seals
Retort pouches and containers	Cooked meat (diced or ground)	A food preservation method involves heating the food product in a hermetically sealed container (e.g., cans, jars, thermoformed containers and retortable pouches).	- Thermally stable (withstand 135 °C) - High gas barrier properties - Good seal integrity - Good toughness, and puncture resistance	- Pouches: outer layer: PET, BOPA, PA, BOPP; middle layer: aluminium foil, PET, PA, PVDC, EVOH; inner layer: HDPE, PP - Thermoformed containers: PP/EVOH, PVDC/PP, CPET - Thermoformed lids: outer layer: PET, PS; middle layer PVDC, EVOH; inner layer: EVA, PP	- Uniform heat treatment - Light weight - Convenient - Long shelf-life	- Cost for machinery and packaging materials - Complex multi-layer films non-recyclable
Thermoformed packaging	Portioned meat; Cooked and processed meat (e.g., luncheon, frankfurter, sausage)	A semi-continuous packaging process which involves heating a semi-rigid film (forming web) to create a moulded base or tray (using vacuum or high pressure). The formed base/tray is then filled with the meat product, and then covered with a lidding material (non-forming web) and vacuum evacuated, sealed and cut into individual packs.	- Good toughness and puncture resistance - Oxygen barrier may be used	- Non-forming web (50–80 µm) and forming web (150–200 µm): LLDPE/Tie/EVOH/PA, ION/Tie/EVOH/PA, LLDPE/Tie/PA, ION/PA, ION/EVA//PVDC/PA, LLDPE/Tie/PA/EVOH/PA/Tie/LLDPE	- High throughput packaging - Reduced packaging volume	- Increased cost and wastage of plastic - Complex multi-layer films non-recyclable - Large pack volume
Modified atmosphere packaging (MAP)	Sub-primal; Portioned cuts; Cooked and processed meat	Meat is packaged or wrapped, vacuum evacuated to remove air and backflushed with a mixture of gases before sealing. The gas mixture can be further controlled through the use of oxygen scavengers as is the case in controlled atmosphere packaging.	- Excellent gas barrier properties	- Forming web/tray: PET, PP, PVDC, EVOH, PVC/PE, PET/PE, PS/EVA/PE, PET/EVA/PE - Non-forming web/lid: LLDPE/Tie/EVOH/PA, ION/Tie/EVOH/PA, LLDPE/Tie/PA, ION/PA, ION/EVA//PVDC/PA, LLDPE/Tie/PA/EVOH/PA/Tie/LLDPE, PVDC/PP/PE, PVDC/PET/PE, PA/PE - Multilayer Film: PA/PE, PA/ION, PA/EVA/PE	- Case ready format - Inhibited colour change and lipid peroxidation in low oxygen gas mixtures - Optimised shelf-life by slowed microbial proliferation	- Cost for gases and machinery - Gas composition needs optimisation - Large pack volume - Complex multi-layer films non-recyclable
Vacuum Packed (VP)	Primal/sub-primal cuts Portioned meat Cooked and processed meat	Meat is preserved by removing air from within the package through vacuum evacuation prior to sealing, slowing proliferation of spoilage microflora.	- Excellent oxygen, moisture, odour and grease barrier properties - High shrinkage - Prevent freezer burn	- Monolayer film: EVOH, PVDC - Multi-layer film: LDPE/EVA/PVDC/EVA/ULDPE, m-LLDPE/EVA/PVDC/EVA/m-LLDPE, m-LLDPE/LLDPE/Tie/EVOH/PA, ION/LLDPE/Tie/EVOH/PA, ION/EVA/LLDPE/PVDC/o-PA	- Case ready format - Optimised shelf-life - Transferable technique to other packaging types	- Loss of preservation once vacuum is lost - Changed meat colour - Complex multi-layer films non-recyclable
Skin packaged	Sub-primal cuts Portioned meat Cooked and processed meat	Skin packaging is a variation of VP. Meat product is placed onto a rigid or flexible barrier material (non-forming web), placed into vacuum chamber and covered with a flexible film (forming web) which is heated, and then vacuum moulded to the product shape.	- Excellent oxygen, moisture, odour and grease barrier properties - Excellent optical properties - High shrinkage	- Non-forming web (50–80 µm) and forming web (150–200 µm): ION/Tie/EVOH/Tie/EVA	- Elegant product presentation - Case ready format - Can be combined with MAP through an additional top forming web to control gas level	- Wrap rage experience - Complex multi-layer films non-recyclable

“/” denotes laminate layering. Abbreviations: vacuum packaging (VP), adhesive polymer layer (Tie), oriented polypropylene (o-PP), expanded polystyrene (EPS), polystyrene (PS), polyethylene terephthalate (PET), crystalline polyethylene terephthalate (CPET), partially neutralised ethylene (meth)acrylic acid (ionomer) (ION), ethylene vinyl alcohol (EVOH), polyvinyl chloride (PVC), polyvinylidene chloride (PVDC), ethylene vinyl acetate (EVA), polyethylene (PE), low density polyethylene (LDPE), linear low density polyethylene (LLDPE), ultra-low density polyethylene (ULDPE), metallocene linear low density polyethylene (m-LLDPE), oriented polyamide (o-PA), biaxially oriented polyamide (BOPA).

## References

[1] Mills J., Donnison A., Brightwell G. (2014). Factors affecting microbial spoilage and shelf-life of chilled vacuum-packed lamb transported to distant markets: A review. Meat Sci..

[2] Schumann B., Schmid M. (2018). Packaging concepts for fresh and processed meat—Recent progresses. Innov. Food Sci. Emerg. Technol..

[3] Food and Agriculture Organisation of the United Nations (2011). Global Food Losses and Food Waste: Extent, Causes and Prevention.

[4] Rosa M.D. (2019). Packaging sustainability in the meat industry. Sustainable Meat Production and Processing.

[5] McMillin K.W. (2017). Advancements in meat packaging. Meat Sci..

[6] Holman B.W., Kerry J.P., Hopkins D.L. (2018). Meat packaging solutions to current industry challenges: A review. Meat Sci..

[7] Ahmed I., Lin H., Zou L., Brody A.L., Li Z., Qazi I.M., Pavase T.R., Lv L. (2017). A comprehensive review on the application of active packaging technologies to muscle foods. Food Control.

[8] Realini C.E., Marcos B. (2014). Active and intelligent packaging systems for a modern society. Meat Sci..

[9] Zhang H., Hortal M., Dobon A., Bermudez J.M., Lara-Lledo M. (2015). The effect of active packaging on minimizing food losses: Life cycle assessment (LCA) of essential oil component-enabled packaging for fresh beef. Packag. Technol. Sci..

[10] Yildirim S., Röcker B., Pettersen M.K., Nilsen-Nygaard J., Ayhan Z., Rutkaite R., Radusin T., Suminska P., Marcos B., Coma V. (2018). Active packaging applications for food. Compr. Rev. Food Sci..

[11] Jafarzadeh S., Jafari S.M., Salehabadi A., Nafchi A.M., Uthaya U.S., Khalil H.A. (2020). Biodegradable green packaging with antimicrobial functions based on the bioactive compounds from tropical plants and their by-products. Trends Food Sci. Technol..

[12] Aliabbasi N., Emam-Djomeh Z., Amighi F., Jafari S.M. (2021). Active food packaging with nano/microencapsulated ingredients. Application of Nano/Microencapsulated Ingredients in Food Products.

[13] Janjarasskul T., Suppakul P. (2018). Active and intelligent packaging: The indication of quality and safety. Crit. Rev. Food Sci. Nutr..

[14] Ribeiro J.S., Santos M.J.M.C., Silva L.K.R., Pereira L.C.L., Santos I.A., da Silva Lannes S.C., da Silva M.V. (2019). Natural antioxidants used in meat products: A brief review. Meat Sci..

[15] (2021). China Statistical Yearbook. Compiled by National Bureau of Statistics of China. https://data.stats.gov.cn/english/easyquery.htm?cn=C01.

[16] Minstry for Primary Industry https://www.mpi.govt.nz/dmsdocument/1018-Livestock-slaughter-statistics-for-sheep-cattle-goats-horses-and-pigs.

[17] Beef + Lamb New Zealand New Season Outlook 2021–22. https://beeflambnz.com/sites/default/files/data/files/New-Season-Outlook-2021-22.pdf.

[18] Nam K.-C., Jo C., Lee M. (2010). Meat products and consumption culture in the East. Meat Sci..

[19] Grunert K.G., Perrea T., Zhou Y., Huang G., Sørensen B.T., Krystallis A. (2011). Is food-related lifestyle (FRL) able to reveal food consumption patterns in non-Western cultural environments? Its adaptation and application in urban China. Appetite.

[20] University of Otago and Ministry of Health, Ministry of Health (2011). A Focus on Nutrition Key Findings of the 2008/09 New Zealand Adult Nutrition Survey.

[21] AUS-MEAT Limited (2015). Handbook of Australian Meat: International Red Meat Manual.

[22] Butler T.I., Morris B.A., Ebnesajjad S. (2013). PE-Based Multilayer Film Structures. Plastic Films in Food Packaging: Materials, Technology, and Applications.

[23] Kropf D.H., Mancini R.A., Devine C., Dikeman M. (2014). Packaging: Modified and controlled atmosphere. Encyclopedia of Meat Sciences.

[24] Kropf D.H., Yancey J.W.S., Yancey E.J., Devine C., Dikeman M. (2014). Packaging: Overwrapping. Encyclopedia of Meat Sciences.

[25] Kropf D.H., Yancey J.W.S., Yancey E.J., Devine C., Dikeman M. (2014). Packaging: Technology and films. Encyclopedia of Meat Sciences.

[26] Lawrence T.E., Kropf D.H., Devine C., Dikeman M. (2014). Packaging: Vacuum. Encyclopedia of Meat Sciences.

[27] McMillin K.W., Belcher J.N., Kerry J.P. (2012). Advances in the packaging of fresh and processed meat products. Advances in Meat, Poultry and Seafood Packaging.

[28] Capita R., Álvarez-González T., Alonso-Calleja C. (2018). Effect of several packaging conditions on the microbiological, physicochemical and sensory properties of ostrich steaks during refrigerated storage. Food Microbiol..

[29] Rodrigues I., Trindade M.A., Palu A.F., Baldin J.C., de Lima C.G., de Alvarenga Freire M.T. (2018). Modified atmosphere packaging for lamb meat: Evaluation of gas composition in the extension of shelf life and consumer acceptance. J. Food Sci. Technol..

[30] Djenane D., Roncalés P. (2018). Carbon monoxide in meat and fish packaging: Advantages and limits. Foods.

[31] Cheah I., Sadat Shimul A., Liang J., Phau I. (2020). Drivers and barriers toward reducing meat consumption. Appetite.

[32] Kim Y.H.B., Ma D., Setyabrata D., Farouk M.M., Lonergan S.M., Huff-Lonergan E., Hunt M.C. (2018). Understanding postmortem biochemical processes and post-harvest aging factors to develop novel smart-aging strategies. Meat Sci..

[33] Fang Z., Zhao Y., Warner R.D., Johnson S.K. (2017). Active and intelligent packaging in meat industry. Trends Food Sci. Technol..

[34] Claus J.R., Sørheim O. (2006). Preserving pre-rigor meat functionality for beef patty production. Meat Sci..

[35] Ge Y., Zhang D., Zhang H., Li X., Fang F., Liang C., Wang Z. (2021). Effect of postmortem phases on lamb meat quality: A physicochemical, microstructural and water mobility approach. Food Sci. Anim. Resour..

[36] Liu H., Hui T., Fang F., Ma Q., Li S., Zhang D., Wang Z. (2021). Characterization and discrimination of key aroma compounds in pre- and postrigor roasted mutton by GC-O-MS, GC E-nose and aroma recombination experiments. Foods.

[37] Xiao X., Hou C., Zhang D., Li X., Ren C., Ijaz M., Hussain Z., Liu D. (2020). Effect of pre- and post-rigor on texture, flavor, heterocyclic aromatic amines and sensory evaluation of roasted lamb. Meat Sci..

[38] Mills J., Horváth K., Reynolds A., Brightwell G. (2018). Farm and abattoir sources of Carnobacterium species and implications for lamb meat spoilage. J. Appl. Microbiol..

[39] Woraprayote W., Malila Y., Sorapukdee S., Swetwiwathana A., Benjakul S., Visessanguan W. (2016). Bacteriocins from lactic acid bacteria and their applications in meat and meat products. Meat Sci..

[40] Bali V., Panesar P.S., Bera M.B., Kennedy J.F. (2016). Bacteriocins: Recent trends and potential applications. Crit. Rev. Food Sci. Nutr..

[41] de Souza de Azevedo P.O., Converti A., Gierus M., de Souza Oliveira R.P. (2019). Application of nisin as biopreservative of pork meat by dipping and spraying methods. Braz. J. Microbiol..

[42] Becerril R., Nerín C., Silva F. (2020). Encapsulation systems for antimicrobial food packaging components: An update. Molecules.

[43] Coban H.B. (2020). Organic acids as antimicrobial food agents: Applications and microbial productions. Bioprocess Biosyst. Eng..

[44] Ouattara B., Simard R.E., Piette G., Bégin A., Holley R.A. (2000). Inhibition of surface spoilage bacteria in processed meats by application of antimicrobial films prepared with chitosan. Int. J. Food Microbiol..

[45] Malhotra B., Keshwani A., Kharkwal H. (2015). Antimicrobial food packaging: Potential and pitfalls. Front. Microbiol..

[46] Dobrucka R., Ankiel M. (2019). Possible applications of metal nanoparticles in antimicrobial food packaging. J. Food Saf..

[47] Chen X., Zhang Y., Yang X., Hopkins D.L., Zhu L., Dong P., Liang R., Luo X. (2019). Shelf-life and microbial community dynamics of super-chilled beef imported from Australia to China. Food Res. Int..

[48] Peck M.W., Goodburn K.E., Betts R.P., Stringer S.C. (2008). Assessment of the potential for growth and neurotoxin formation by non-proteolytic Clostridium botulinum in short shelf-life commercial foods designed to be stored chilled. Trends Food Sci. Technol..

[49] Adzitey F., Nurul H. (2011). Pale soft exudative (PSE) and dark firm dry (DFD) meats: Causes and measures to reduce these incidences-a mini review. Int. Food Res. J..

[50] Castejón D., García-Segura J.M., Escudero R., Herrera A., Cambero M.I. (2015). Metabolomics of meat exudate: Its potential to evaluate beef meat conservation and aging. Anal. Chim. Acta.

[51] Otoni C.G., Espitia P.J.P., Avena-Bustillos R.J., McHugh T.H. (2016). Trends in antimicrobial food packaging systems: Emitting sachets and absorbent pads. Food Res. Int..

[52] Agrimonti C., White J.C., Tonetti S., Marmiroli N. (2019). Antimicrobial activity of cellulosic pads amended with emulsions of essential oils of oregano, thyme and cinnamon against microorganisms in minced beef meat. Int. J. Food Microbiol..

[53] Zhang R., Yoo M.J.Y., Farouk M.M. (2019). Quality and acceptability of fresh and long-term frozen in-bag dry-aged lean bull beef. J. Food Qual..

[54] Zhang R., Yoo M.J., Realini C.E., Staincliffe M., Farouk M.M. (2021). In-bag dry- *vs.* wet-aged lamb: Quality, consumer acceptability, oxidative stability and *in vitro* digestibility. Foods.

[55] Shi Y., Zhang W., Zhou G. (2020). Effects of different moisture-permeable packaging on the quality of aging beef compared with wet aging and dry aging. Foods.

[56] Zhang R., Yoo M.J.Y., Farouk M.M. (2021). Oxidative stability, proteolysis, and *in vitro* digestibility of fresh and long-term frozen stored in-bag dry-aged lean beef. Food Chem..

[57] Zhang R., Yoo M.J.Y., Ross A.B., Farouk M.M. (2022). Mechanisms and strategies to tailor dry-aged meat flavour. Trends Food Sci. Technol..

[58] Lee H.J., Choe J., Kim K., Oh J., Lee D., Kwon K., Choi Y., Jo C. (2017). Analysis of low-marbled Hanwoo cow meat aged with different dry-aging methods. Asian-Australasian J. Anim. Sci..

[59] Berger J., Kim Y.H.B., Legako J.F., Martini S., Lee J., Ebner P., Zuelly S.M.S. (2018). Dry-aging improves meat quality attributes of grass-fed beef loins. Meat Sci..

[60] Zhang R., Ross A.B., Yoo M.J.Y., Farouk M.M. (2021). Use of Rapid Evaporative Ionisation Mass Spectrometry fingerprinting to determine the metabolic changes to dry-aged lean beef due to different ageing regimes. Meat Sci..

[61] Choe J.H., Stuart A., Kim Y.H.B. (2016). Effect of different aging temperatures prior to freezing on meat quality attributes of frozen/thawed lamb loins. Meat Sci..

[62] Gudjónsdóttir M., Gacutan M.D., Mendes A.C., Chronakis I.S., Jespersen L., Karlsson A.H. (2015). Effects of electrospun chitosan wrapping for dry-ageing of beef, as studied by microbiological, physicochemical and low-field nuclear magnetic resonance analysis. Food Chem..

[63] Hobbs J.E. (2021). The COVID-19 pandemic and meat supply chains. Meat Sci..

[64] Fanelli R.M. (2021). Changes in the Food-Related Behaviour of Italian Consumers during the COVID-19 Pandemic. Foods.

[65] Domínguez R., Barba F.J., Gómez B., Putnik P., Kovačević D.B., Pateiro M., Santos E.M., Lorenzo J.M. (2018). Active packaging films with natural antioxidants to be used in meat industry: A review. Food Res. Int..

[66] Utrera M., Morcuende D., Estévez M. (2014). Fat content has a significant impact on protein oxidation occurred during frozen storage of beef patties. LWT.

[67] Holman B.W., Coombs C.E., Morris S., Kerr M.J., Hopkins D.L. (2017). Effect of long term chilled (up to 5 weeks) then frozen (up to 12 months) storage at two different sub-zero holding temperatures on beef: 1. Meat quality and microbial loads. Meat Sci..

[68] Gómez-Estaca J., López-de-Dicastillo C., Hernández-Muñoz P., Catalá R., Gavara R. (2014). Advances in antioxidant active food packaging. Trends Food Sci. Technol..

[69] Granato D., Nunes D.S., Barba F.J. (2017). An integrated strategy between food chemistry, biology, nutrition, pharmacology, and statistics in the development of functional foods: A proposal. Trends Food Sci. Technol..

[70] Souza V.G.L., Rodrigues P.F., Duarte M.P., Fernando A.L. (2018). Antioxidant migration studies in chitosan films incorporated with plant extracts. J. Renew. Mater..

[71] Arrieta M.P., Garrido L., Faba S., Guarda A., Galotto M.J., López de Dicastillo C. (2020). Cucumis metuliferus fruit extract loaded acetate cellulose coatings for antioxidant active packaging. Polymers.

[72] Surendhiran D., Li C., Cui H., Lin L. (2020). Fabrication of high stability active nanofibers encapsulated with pomegranate peel extract using chitosan/PEO for meat preservation. Food Packag. Shelf Life.

[73] Hassanzadeh P., Tajik H., Rohani S.M.R., Moradi M., Hashemi M., Aliakbarlu J. (2017). Effect of functional chitosan coating and gamma irradiation on the shelf-life of chicken meat during refrigerated storage. Radiat. Phys. Chem..

[74] Olszewska M.A., Gędas A., Simões M. (2020). Antimicrobial polyphenol-rich extracts: Applications and limitations in the food industry. Food Res. Int..

[75] Lin L., Liao X., Cui H. (2019). Cold plasma treated thyme essential oil/silk fibroin nanofibers against *Salmonella typhimurium* in poultry meat. Food Packag. Shelf Life.

[76] Pavli F., Argyri A.A., Skandamis P., Nychas G.-J., Tassou C., Chorianopoulos N. (2019). Antimicrobial activity of oregano essential oil incorporated in sodium alginate edible films: Control of *Listeria monocytogenes* and spoilage in ham slices treated with high pressure processing. Materials.

[77] Carina D., Sharma S., Jaiswal A.K., Jaiswal S. (2021). Seaweeds polysaccharides in active food packaging: A review of recent progress. Trends Food Sci. Technol..

[78] Cui H., Bai M., Li C., Liu R., Lin L. (2018). Fabrication of chitosan nanofibers containing tea tree oil liposomes against *Salmonella* spp. in chicken. LWT.

[79] Zhao Y., Teixeira J.S., Saldaña M.D., Gänzle M.G. (2019). Antimicrobial activity of bioactive starch packaging films against *Listeria monocytogenes* and reconstituted meat microbiota on ham. Int. J. Food Microbiol..

[80] Bolumar T., LaPeña D., Skibsted L.H., Orlien V. (2016). Rosemary and oxygen scavenger in active packaging for prevention of high-pressure induced lipid oxidation in pork patties. Food Packag. Shelf Life.

[81] Cardoso G.P., Dutra M.P., Fontes P.R., Ramos A.d.L.S., de Miranda Gomide L.A., Ramos E.M. (2016). Selection of a chitosan gelatin-based edible coating for color preservation of beef in retail display. Meat Sci..

[82] Hutter S., Rüegg N., Yildirim S. (2016). Use of palladium based oxygen scavenger to prevent discoloration of ham. Food Packag. Shelf Life.

[83] Ribeiro-Santos R., de Melo N.R., Andrade M., Azevedo G., Machado A.V., Carvalho-Costa D., Sanches-Silva A. (2018). Whey protein active films incorporated with a blend of essential oils: Characterization and effectiveness. Packag. Technol. Sci..

[84] Borzi F., Torrieri E., Wrona M., Nerín C. (2019). Polyamide modified with green tea extract for fresh minced meat active packaging applications. Food Chem..

[85] Yordshahi A.S., Moradi M., Tajik H., Molaei R. (2020). Design and preparation of antimicrobial meat wrapping nanopaper with bacterial cellulose and postbiotics of lactic acid bacteria. Int. J. Food Microbiol..

[86] Behbahani B.A., Shahidi F., Yazdi F.T., Mortazavi S.A., Mohebbi M. (2017). Use of Plantago major seed mucilage as a novel edible coating incorporated with Anethum graveolens essential oil on shelf life extension of beef in refrigerated storage. Int. J. Biol. Macromol..

[87] Kaewprachu P., Osako K., Benjakul S., Rawdkuen S. (2015). Quality attributes of minced pork wrapped with catechin–lysozyme incorporated gelatin film. Food Packag. Shelf Life.

[88] Radha Krishnan K., Babuskin S., Rakhavan K., Tharavin R., Azhagu Saravana Babu P., Sivarajan M., Sukumar M. (2015). Potential application of corn starch edible films with spice essential oils for the shelf life extension of red meat. J. Appl. Microbiol..

[89] Catarino M.D., Alves-Silva J.M., Fernandes R.P., Gonçalves M.J., Salgueiro L.R., Henriques M.F., Cardoso S.M. (2017). Development and performance of whey protein active coatings with *Origanum virens* essential oils in the quality and shelf life improvement of processed meat products. Food Control.

[90] Kalem I.K., Bhat Z.F., Kumar S., Noor S., Desai A. (2018). The effects of bioactive edible film containing *Terminalia arjuna* on the stability of some quality attributes of chevon sausages. Meat Sci..

[91] Shahbazi Y., Shavisi N. (2018). A novel active food packaging film for shelf-life extension of minced beef meat. J. Food Saf..

[92] Li L., Wang H., Chen M., Jiang S., Cheng J., Li X., Zhang M., Jiang S. (2020). Gelatin/zein fiber mats encapsulated with resveratrol: Kinetics, antibacterial activity and application for pork preservation. Food Hydrocoll..

[93] Byun Y., Kim Y.T., Han J.H. (2014). Bioplastics for food packaging: Chemistry and physics. Innovations in Food Packaging.

[94] Schyns Z.O.G., Shaver M.P. (2021). Mechanical Recycling of Packaging Plastics: A Review. Macromol. Rapid Commun..

[95] Zhong Y., Godwin P., Jin Y., Xiao H. (2020). Biodegradable polymers and green-based antimicrobial packaging materials: A mini-review. Adv. Ind. Eng. Polym. Res..

[96] Mostafavi F.S., Zaeim D. (2020). Agar-based edible films for food packaging applications—A review. Int. J. Biol. Macromol..

[97] Molinaro S., Cruz-Romero M., Sensidoni A., Morris M., Lagazio C., Kerry J.P. (2015). Combination of high-pressure treatment, mild heating and holding time effects as a means of improving the barrier properties of gelatin-based packaging films using response surface modeling. Innov. Food Sci. Emerg. Technol..

[98] Hassan M.M., Le Guen M.J., Tucker N., Parker K. (2019). Thermo-mechanical, morphological and water absorption properties of thermoplastic starch/cellulose composite foams reinforced with PLA. Cellulose.

[99] Umaraw P., Munekata P.E., Verma A.K., Barba F.J., Singh V., Kumar P., Lorenzo J.M. (2020). Edible films/coating with tailored properties for active packaging of meat, fish and derived products. Trends Food Sci. Technol..

[100] Gheorghita R., Gutt G., Amariei S. (2020). The Use of Edible Films Based on Sodium Alginate in Meat Product Packaging: An Eco-Friendly Alternative to Conventional Plastic Materials. Coatings.

[101] Nilsen-Nygaard J., Fernández E.N., Radusin T., Rotabakk B.T., Sarfraz J., Sharmin N., Sivertsvik M., Sone I., Pettersen M.K. (2021). Current status of biobased and biodegradable food packaging materials: Impact on food quality and effect of innovative processing technologies. Compr. Rev. Food Sci. Food Saf..

[102] Zubair M., Ullah A. (2020). Recent advances in protein derived bionanocomposites for food packaging applications. Crit. Rev. Food Sci. Nutr..

[103] Ajitha A.R., Aswathi M.K., Maria H.J., Izdebska J., Thomas S., Kim J.K., Thomas S., Saha P. (2016). Multilayer Polymer Films. Multicomponent Polymeric Materials.

[104] Anukiruthika T., Sethupathy P., Wilson A., Kashampur K., Moses J.A., Anandharamakrishnan C. (2020). Multilayer packaging: Advances in preparation techniques and emerging food applications. Compr. Rev. Food Sci. Food Saf..

[105] Hassan M.M., Tiwari A. (2017). Antimicrobial coatings for textiles. Handbook of Antimicrobial Coatings.

[106] Bastarrachea L.J., Wong D.E., Roman M.J., Lin Z., Goddard J.M. (2015). Active Packaging Coatings. Coatings.

[107] Morris M.A., Padmanabhan S.C., Cruz-Romero M.C., Cummins E., Kerry J.P. (2017). Development of active, nanoparticle, antimicrobial technologies for muscle-based packaging applications. Meat Sci..

[108] Han J.W., Ruiz-Garcia L., Qian J.P., Yang X.T. (2018). Food packaging: A comprehensive review and future trends. Compr. Rev. Food Sci. Food Saf..

[109] Sherman L. (2018). Scion Seeks to Understand China’s Food Contact Requirements.

[110] Skaggs K.C., Nielsen C.R. (2018). Australia/New Zealand: Keeping in Contact with Food Packaging.

[111] Miller J. (2018). Australia and New Zealand will not Regulate Food Packaging.

